# Editorial: Food safety in the context of One Health: current trends, challenges and perspectives

**DOI:** 10.3389/fmicb.2026.1894811

**Published:** 2026-07-14

**Authors:** Daniel Ekhlas, Yosra A. Helmy, Federica Savini, Arturo B. Soro, Elena-Alexandra Alexa

**Affiliations:** 1 AIRLAB, Climate and Health Group, Climate, Air Pollution, Nature and Urban Health (CANU) Program, Barcelona Institute for Global Health, Barcelona, Spain; 2 Department of Veterinary Science, Martin-Gatton College of Agriculture, Food, and Environment, University of Kentucky, Lexington, KY, United States; 3 Department of Veterinary Medical Sciences, Alma Mater Studiorum, University of Bologna, Ozzano dell'Emilia, Bologna, Italy; 4 Departament de Nutrició, Ciències de l'Alimentació i Gastronomia, Facultat de Farmàcia i Ciències de l'Alimentació, Campus de l'Alimentació de Torribera, University of Barcelona, Barcelona, Spain; 5 School of Food Science and Environmental Health, Technological University Dublin, Dublin, Ireland; 6 Centre for Antimicrobial Resistance and One-Health Research, Technological University Dublin, Dublin, Ireland

**Keywords:** antimicrobial resistance, food safety, One Health, sequencing, transmission

As the global population is expected to reach 9.7 billion by 2050, we are facing new challenges in meeting the rising global demand but also in addressing inequalities in accessing safe food and clean water. The impact of climate change and associated extreme weather events, including droughts and flooding, further limits our ability to ensure access to these essential resources. For example, recurrent heavy rainfall events and flooding can lead to the contamination of water bodies, which in turn results in the dissemination of pathogenic and antimicrobial resistant *Escherichia coli, Campylobacter, Salmonella*, and enteric viruses into agricultural fields and farming environments. Moreover, rising temperatures driven by climate change may promote the growth of *Vibrio* species in coastal zones and estuaries, thereby affecting aquaculture production. On the other hand, consumers have shifted their food consumption patterns, with more sustainable practices being adopted but at the same time ensuring food safety remains a critical concern. Current methodologies face new challenges due to these dynamics, for which the One Health concept has evolved from a simple guiding principle into a widely recognized framework. These challenges, together with recent positive developments, create new opportunities for international collaboration and innovative research and policy making to improve surveillance, prevention, and control strategies in food. Therefore, the aim of this Research Topic is to explore multifaceted strategies to ensure food safety within the One Health framework while focusing on sustainable food production, the management of microbiomes in agriculture, and the development of new decontamination technologies. Within this Research Topic, 14 articles have been published, incorporating surveillance and detection, transmission pathways, antimicrobial resistance (AMR), and innovative intervention and control strategies for foodborne pathogens.

Among these contributions, particular attention has been given to fruits and vegetables. In this context, Tenea and Angamarca reported the first complete genome of *Staphylococcus xylosus* FFCShyA4 with a predicted human pathogenicity of 98.2%. The multidrug resistant strain was isolated from the exocarp of Guatemalan avocados, in which intensive genomic analysis of the strain confirmed the carriage of multiple antimicrobial resistance genes (ARGs) conferring resistance to β-lactams, macrolides, fluoroquinolones, tetracyclines, and lincosamides. The presence of 133 mobile genetic elements (MGEs) additionally highlighted the potential for the mobility of these ARGs via horizontal gene transfer. Similarly, Korsak et al., *Cronobacter* spp. detected in 26 of 251 different food samples, including raw milk, powdered milk, infant formula, dried herbs and spices, dried teas, and raw vegetables, as part of a surveillance study conducted in Poland. The isolated *Cronobacter* strains were classified into five species, with *C. sakazakii* showing the highest prevalence. One strain *C. sakazakii* MK_10, isolated from dried oregano, was fully sequenced as the first *C. sakazakii* sequenced strain in Poland. The strain carried multiple MGEs with one MGE carrying resistance determinants against heavy metal (copper homeostasis and silver resistance island; CHASRI). Both studies underscore the potential for discovery and the necessity of new surveillance approaches for novel foodborne pathogens.

Branstad-Spates et al. investigated the susceptibility of protein-rich pulse crops, including chickpeas, peas, and lentils to aflatoxigenic *Aspergillus flavus* (AF-70 GFP) infection in a 10-day *in vitro* study. While aflatoxin production was lower in pulse crops compared to controls (corn), elevated concentrations of cyclopiazonic acid and α-aflatrem were observed. The study states that further research of fungal infections and mycotoxin production of pulse crops and pulse crop-derived products is required, as crops on field and under storage conditions may show different susceptibility profiles.

In contrast, Gomes et al. investigated the effects of using secondary- and tertiary-treated wastewater for irrigation of lettuce plants on the transmission of drug-resistant bacteria and ARGs. While using extended-spectrum β-lactamase (ESBL) and non-ESBL *Escherichia coli* as indicators for bacterial transmission, quantification was only possible in lettuce crops irrigated with secondary-treated water due to the limit of detection. Targeted quantitative PCR (16S rRNA genes, *bla*_CTX−*M*−1_, *bla*_TEM_, *sul1*, and *tetA*) showed that secondary-treated water contained higher absolute abundances of ARGs compared to tertiary-treated wastewater. Thus, fractions of ARGs were transmitted and detected in lettuce crops after irrigation with secondary- and tertiary-treated wastewater (6% and 4%, respectively). This highlights the importance of irrigation water quality to prevent the transmission and contamination of crops with antimicrobial resistant microorganisms. This additionally applies for the transmission of foodborne viruses via irrigation water. Accordingly, Casado-Martín et al. took a wastewater-based epidemiological approach for monitoring enteric viruses, including human noroviruses, human astroviruses, rotaviruses, and hepatitis A and E viruses, often underreported in populations. The study conducted from 2020 to 2021 showed promising results for early detection and monitoring of enteric virus outbreaks. A similar approach may be applicable for monitoring viral contamination in irrigation water. Likewise, Ferri et al. monitored the same enteric viruses in unpasteurized caprine milk in Italy. In total, 59 of 383 milk samples (15.4%) tested positive for at least one viral pathogen, emphasizing the importance of milk processing via pasteurization for viral pathogens inactivation. Besides, the consumption of unpasteurized milk or dairy products can also lead to infection with bacterial diseases such as brucellosis. Mascolo et al. investigated prevention methods for brucellosis outbreaks and reinfection by conducting a retrospective cohort study of 222 outbreaks between 2016 and 2021. The findings indicated that stamping out, together with strengthened biosecurity measures, reduced reinfection risk by 80%, helping to protect both consumer safety and animal health.

Further focusing on meat and other animal products, a case study conducted by Lu et al. reported a botulism outbreak caused by *Clostridium botulinum* subtype A2 contamination in pickled eggs in China. Ten strains were isolated from pickled eggs and patient feces. Analyzed for ARGs and virulence genes via real-time PCR, mouse bioassay, and whole-genome sequencing revealed that all strains belonged to the MLST type ST193 and carried the *cfr, spw*, and *vat* ARGs. Moreover, Chowdhury et al. tested thousands of food samples together with environmental samples from food processing environments, and diarrheal stools from hospitalized patients for *Proteus mirabilis* between 2021 and 2024 in four states of Northeast India. *P. mirabilis* was predominantly detected in environmental samples (3.2%), but also in food samples with varying prevalence depending on the state. Isolated *P. mirabilis* strains exhibited high prevalence of resistance to quinolone/fluroquinolones, nalidixic acid, erythromycin, tetracycline, and doxycycline, while meropenem resistance differed depending on whether foods were state-specific.

Similarly, Habib et al. investigated AMR and MGEs in *E. coli* strains isolates from 253 frozen whole broiler carcasses. In total, 90 isolates were tested using conventional antimicrobial susceptibility testing, while 33 of these isolates were further analyzed using whole-genome sequencing. Of 253 frozen broiler carcasses, 248 tested positive for *E. coli* resistant to ampicillin (52.2%) and tetracycline (35.6%), while 68.9% of isolates exhibited multidrug resistance. Sequencing results confirmed the strong presence of *bla*_CTX−*M*−55_ and *bla*_CTX−*M*−8_ ESBL resistance genes in co-occurrence with *qnrS1* and *qnrB19*, conferring resistance to fluoroquinolones, and the presence of these ARGs on conjugative plasmids. Comparably, Papoula-Pereira et al. quantified *Campylobacter* spp. and Enterobacterales on broiler carcasses in commercial abattoir in England and after applying intervention methods (hot water immersion/ultrasound intervention) during processing. *Campylobacter* isolates predominantly exhibited resistance to tetracycline (45%), nalidixic acid (41%), and ciprofloxacin (39%), with 2.7% being multidrug resistant. Intervention methods were able to reduce *Campylobacter* and Enterobacterales levels by 0.85 and 0.82 log_10_, respectively. Similar observations for AMR profiles of *C. jejuni* have been made by Yang et al., which screened 18 *C. jejuni* isolates from human stool and 29 isolates from raw poultry meat in Hangzhou, China. Majority exhibited multidrug resistance while CC-443 and CC-574 clonal complexes being predominant. Additional intervention methods as described by Papoula-Pereira et al., such as phage-based biocontrol using a trained three-phage cocktail for the control of *Salmonella* and *E. coli* levels in chicken filets has been investigated under laboratory conditions by Berhilevych et al. The phage-based treatment was able to achieve reductions of 1.56 log10 CFU/mL and 1.48 log_10_ CFU/mL for *Salmonella* BfR 20-SA00418 and *E. coli* 19/302/1/A after for 72 h storage at 4 °C ± 0.5 °C, suggesting the potential of phage-based biocontrol as an alternative for enhancing food safety in poultry meat production. Further challenges for these intervention methods may also present the association of foodborne pathogens with microplastics. Ortega-Sanz and Rajkovic investigated associations of *C. jejuni* contamination and AMR with microplastics. For the *in vitro* study, five *C. jejuni* strains from different origins were incubated for up to 24–72 h, together with 5 mm discs of biaxially oriented polyethylene terephthalate (BOPET), usually used for food packaging. Interestingly, while biofilm formation on microplastic discs contributed to the persistence of *C. jejuni* strains, biofilm-derived strains exhibited a higher susceptibility to antibiotics.

Overall, the articles within the Research Topic cover a great variety of different bacterial, fungal, and viral pathogens as well as various food and food products, and underscore the need for more surveillance, exploratory, and intervention studies for the prevention of foodborne disease and AMR transmission in the perspective of One Health.

